# Rigour versus the need for evidential diversity

**DOI:** 10.1007/s11229-021-03368-1

**Published:** 2021-11-01

**Authors:** Nancy Cartwright

**Affiliations:** 1 Durham University, Durham, UK; 2 University of California at San Diego, San Diego, CA USA

**Keywords:** Singular causality, Mixed methods, Methodological diversity, Evidential diversity, Theory of change, Causal processes, Rigour, RCT

## Abstract

This paper defends the need for evidential diversity and the mix of methods that that can in train require. The focus is on causal claims, especially ‘singular’ claims about the effects of causes in a specific setting—either what will happen or what has happened. I do so by offering a template that categorises kinds of evidence that can support these claims. The catalogue is generated by considering what needs to happen for a causal process to carry through from putative cause at the start to the targeted effect at the end. The usual call for mixed methods focusses on a single overall claim and argues that we increase certainty by the use of different methods with compensating strengths and weaknesses. My proposals instead focus on the evidence that supports the great many subsidiary claims that must hold if the overall one is to be true. As is typical for singular causal claims, the mix of methods that will generally be required to collect the kinds of evidence I urge will usually have little claim to the kind of rigour that is now widely demanded in evidencing causal claims, especially those for policy/treatment effectiveness. So I begin with an exploration of what seems to be intended by ‘rigour’ in such discussions, since it is seldom made clear just what makes the favoured methods especially rigorous. I then argue that the emphasis on rigour can be counterproductive. Rigour is often the enemy of evidential diversity, and evidential diversity—lots of it—can make for big improvements in the reliability of singular causal predictions and post hoc evaluations. I illustrate with the paragon of rigour for causal claims, randomised controlled trials (RCTs), rehearsing at some length what they can and cannot do to make it easier to assess the importance of rigour in warranting singular causal claims.

## What to expect

This paper defends the need for evidential diversity and the mix of methods that that can in train require. The focus is on causal claims, especially ‘singular’ claims about the effects of causes in a specific setting—either what will happen or what has happened there. Or, to put this more carefully since I will be dealing with multivalued variables: I focus on ‘what a cause *will contribute* or *has contributed*’. I defend the need for evidential diversity by offering a template that categorises kinds of evidence needed for tracing a causal process from cause to effect.

As with most methodologies for singular causal hypotheses, the mix of methods that it would take to do what I urge has little claim to the kind of rigour that is now widely demanded in evidencing causal hypotheses, especially those for policy/treatment effectiveness. This can seem like a damming charge against this and almost any methodologies using mixed methods. But I argue that it will not seem so damming if we look seriously at what rigour might amount to for methods that are praised for being most rigorous in establishing causal claims. This is especially so considering what conclusions can be rigorously established by these methods if successfully implemented and how many things often not mentioned can get in the way of successful implementation.

I aim for this paper to make a number of interconnected contributions.

First, I provide a *catalogue of different kinds of information that can serve as evidence for singular causal claims*.

Second I provide a *defence that these are evidence*. The catalogue is based in a widely acceptable account of what it takes for a casual process to carry through from the intervention at the start to the targeted effect at the end. Everything I say to back up their claim to be evidence is straightforward. Each different kind of evidence has an advocate in some current school of thought about causality, each fits with common sense, each has solid philosophical credentials and each is readily acknowledged by researchers across the natural and social sciences. Yet this catalogue is different from the currently dominant ways of approaching the issue and, once all the different types are put together, is richer than the offerings of other minority approaches.

Third, we are told across the social and economic sciences and especially by the evidenced-based medicine/policy movements (EBM, EBP) that randomised controlled trials (RCTs) are the most rigorous way to support causal conclusions. But I have failed to find anything like a characterisation of what is meant by ‘rigour’, nor, in train, a defence of why what might be meant deserves the honorific ‘rigour’, nor a proper argument that RCTs indeed meet the criteria supposed in the characterisation. As Angus Deaton, who won the 2015 Nobel prize in economics for his analysis of consumption, poverty, and welfare, remarks under the heading *RCTs are rigorous and scientific*: ‘This rhetoric is rarely if ever justified. The adjectives [rigorous, scientific] are used as code words for RCTs. Frequently so.’ (Deaton, 2020, p 8.) Here I offer a *characterisation of what could be meant by rigour* that makes sense, as best I can, of the claims made on behalf of RCTs to be rigorous. The characterisation is based on an analysis of what RCTs seem uniquely able to provide plus an ear to what is said in their defence, especially in economics where rigour is traditionally both highly prized and claimed.

An anonymous referee suggests dropping this analysis on the grounds that it is already well-known, including from my own paper with Angus Deaton (Deaton & Cartwright, 2018). I take that to be an advantage. It shows I am not making up an account of what RCTs can do just to fit my purposes here. At any rate what has not been done before so far as I know is to use this analysis to extract an account of what might be meant by claiming that RCTs are the most rigorous way to establish causal claims.

Fourth, I argue that, after all, *even well-conducted RCTs are often unable to live up to the demands of rigour* that I have identified. This can seem to be a cheat. First, based in part on an analysis of what RCTs seem uniquely able to provide, I say that rigour requires X, Y and Z. Then I say that, after all, often even well-conducted RCTs cannot supply X, Y and Z. I believe though that when we get to the details, it will not seem a cheat. The defence of RCTs depends on what randomisation can supply that is otherwise missing from statistical studies. But the problems are with what can happen post randomisation, where the widely recommended practice of blinding at multiple points cannot control for all that can go wrong.^1^

Fifth, tying this all together. The kinds evidence that appear in my catalogue cannot rigorously establish the singular causal claim they argue for in the sense of rigour I identify, even if a great deal of such evidence is available. But then often RCTs cannot do so either and RCTs are widely taken as the gold standard in rigour in establishing causal claims. This seems to me to argue for the conclusion that *rigour is an overvalued virtue in evaluating evidence for causal claims* and that we should admit that though the evaluation of what the evidence shows can be done carefully and critically, *the call for evidence that rigorously establishes our conclusions is misplaced*.

Sixth, finally all this together provides a *shift in perspective about the use of mixed methods for supporting causal conclusions.* The usual call for mixed methods focusses on a single overall claim and argues that we increase certainty by the use of different methods with compensating strengths and weaknesses. My proposal instead focuses on the evidence that supports the great many subsidiary claims that must hold if the overall one is to be true. The template that I offer catalogues a variety of different kinds of evidence that do this job.

## Rigour, RCTs and pTocs

The call for rigour in science is widespread. The social sciences are no exception. Consider education for example. The US National Research Council’s, 2002 report, *Scientific research in education* claims that.

- *…*what unites scientific inquiry is the primacy of empirical test of conjectures and formal hypotheses using well-codified observation methods and *rigorous designs*, and subjecting findings to peer review. (p. 51. ital. added)

This view is not peculiar to the US. Here, for example, is what the Ministry of Education in New Zealand has to say:

- Scientific explanations are accepted as reliable only when they have been subjected to *rigorous testing*.^2^

Rigour has substantial charm as a road to securing objectivity and making results more certain. But it has a substantial drawback: you can’t do much with it. The kinds of results that can be secured rigorously cannot support much on their own. To build secure support for the kinds of outcomes that matter both in practice and in principle, rigorous designs must be woven together with a vast amount of other scientific work that uses a panoply of different methods—‘a tangle of science’.

I am going to illustrate with the case of randomized controlled trials (*RCTs*) as my exemplar of a method for warranting causal hypotheses that is widely praised for its rigour. Ever since the ‘credibility revolution’ in economics and the spreading influence of JPAL^3^ in development economics, topped off by the Nobel prize for Abhijit Banerjee, Esther Duflo, and Michael Kremer in 2019 for what the Nobel announcement describes as ‘their new experiment-based approach [that] has transformed development economics’,^4^ there has been a marked restriction in what ‘best practice’ counts as good evidence for causal hypotheses in economics—and elsewhere in the human sciences. In economics, when it comes to assessing causal claims, evidence from RCTs is most prized, that from instrumental variables models and natural experiments less so and that provided by economic theory is deemed highly suspicious, along with both theoretical and structural modelling. Causal process tracing, qualitative comparative analysis (QCA) and ethnographic and other ‘qualitative’ methods usually don’t even get a mention.

But economics is not special here. Emphasis is put on RCTs everywhere you hear the cry for ‘evidence-based policy’. And they are still gold standard at the very influential Campbell Collaboration (whose motto is ‘Better evidence for a better world’):

Campbell reviews are intended to summarize both the *best* evidence available about the effects of the focal intervention(s) and *all* the evidence that provides credible estimates of those effects. The critical feature of the research methods in this regard is the ability of the basic design to yield an unbiased estimate of the effects on the target outcomes relative to a defined counterfactual condition, that is, the internal validity of the research design... With rare exceptions, the best evidence by this standard is provided by randomized controlled trials (RCTs). (Campbell 2014, p. 9, ital. orig.)

Generally this kind of restriction is a mistake. Privileging methods, any methods, narrows the scope of what science is up to. It restricts the questions that can get asked—a common complaint, and it cements in a metaphysics, a metaphysics that may or may not be appropriate to the context under study.

What an RCT by itself can give you is, as the Campbell quote notes, an unbiased estimate. What can thus be estimated is the average treatment effect (*ATE*) in the population studied. Causal claims of interest are seldom about the populations enrolled in a study but about other populations—or individual units—elsewhere. RCTs can be part of an evidence base for those, especially if you have a lot of information about the relations between the causal principles at work in the new population and the arrangement of causal factors that obtains there compared with those of the study population. That already creates a need for a good mix of methods. And at that you will still probably only be able to estimate some kind of average whereas you are frequently interested in causal predictions about a single unit—this client, this pupil, this village, this school, this state.

I propose a different, more direct way of warranting a causal hypothesis about a specific population or a specific unit: by building a special kind of theory of change—a causal-process-tracing theory of change (*pToC*) for that population or that unit.

Theories of change (*ToCs*) are by now standard fare for predicting, implementing and evaluating the effects of policy interventions. They map the steps by which the cause is supposed to produce the effect. The special causal-process-tracing versions of these that I advocate contain much more information than standard ToCs and are thereby much more useful.

Why is this such a good way to provide warrant? It is such a good way because the pToC mirrors what actually is supposed to happen on the ground once a cause has fired. It tells what the process is by which the changes the cause initiates eventuate in effects down the line and what those effects are. Whether you are in a position to learn these facts or make an effort to do so, the facts that are represented in the pToC are the ones that matter to whether the effect will be achieved or not.

So, my claim is that when it comes to warranting effectiveness claims, we need a large variety of methods to secure the information depicted in the pToC. Not many of these methods will come up to the standards that seem required to earn the honorific ‘rigorous’. Yet we cannot do without them.

I shall defend this in four stages. First I will review just what is so special about RCTs in order to identify what could plausibly be meant by claiming they are the ‘most rigorous methods’ for warranting causal claims. This brings into focus just what question an RCT can rigorously answer and what restrictive metaphysical assumptions we must presuppose to defend the RCT’s claim to rigour. Second I shall point out how little we can do with the answers we can get this way without combining them with a great deal of other information that we can only get by using a variety of different methods that collectively have no such claims to rigor. Third I shall urge that for evidencing a singular causal hypothesis we should produce a causal-process-tracing theory of change, showing the sequence of steps by which the cause is to produce the effect, including the causal principles under which each step generates the next and the interactive factors and derailers that may operate at each step. This provides us with a catalogue of varieties of evidence types relevant to evaluating whether the process can carry through start to finish. Fourth I shall argue that providing the evidence detailed in this kind of theory of change always requires a broad mix of methods, including a good dollop of middle-level economic and social theory and common knowledge.

One defence of mixed methods involves concerns that no single method can ever provide really good evidence for a hypothesis. You need a variety of different methods that might fail in different ways. That requires mixed methods. As I have noted, my argument is different. I point out not the need for different methods applied directly to one and the same causal hypothesis but the need to warrant the hypothesis by warranting a large number of different subsidiary hypotheses which are of very different kinds and thus require a variety of different methods.

## What’s so rigorous about an RCT and what good does it do you?^5^

We are often told that what’s so good about RCTs is that only they can control for statistical bias. How does that make them rigorous: Why does controlling for statistical bias earn a method for evidencing causal claims the honorific label ‘rigorous’, and what sense can be given to ‘rigour’ to make this claim to rigour credible? To answer that we need to understand what role ‘controlling for statistical bias’ plays in helping RCTs provide evidence for causal conclusions. And to do that, we need to understand just what is meant by ‘controlling for statistical bias’, just how RCT methods achieve that, and just what kind of conclusion is evidenced.

First you need the context. Suppose you want to know about whether a specific factor C causes a targeted outcome E in members of a given population P and you are in the happy situation of knowing that in P the expectation of E is greater with C than without C (Exp(E/C) > Exp(E/-C)). Still, we know that goes little way to ensuring a causal connection because of the possibility of statistical bias (statistical as opposed to bias in the sense of prejudice), that is, put loosely, C might be ‘correlated with’ other causal factors for E—the dependence of E on C might be statistically biased—and *that* could be what’s responsible for the (spurious) association of C and E. If there were no such bias, you could infer causation. Well, you could—supposing a lot of metaphysics, like every event has a causal explanation, that for a given event in a given population there is a given set of features that can cause it, and that there is a probability measure over the features in what is called the ‘potentuial outcome equation’ (*POE*) for the population studied.

The relevant result can be proven mathematically, and I think the metaphysics is clearer from the mathematical proof. A standard proof starts with a *potential outcomes equation* and ends with the conclusion that Exp(E/C)-Exp(E/-C) (in an RCT this will be the expectation of E in the test arm minus the expectation of E in the control arm) is an *unbiased estimate* of the *average treatment effect* of C on E. I’ll sketch this briefly. For much more discussion see Deaton and Cartwright (2018).

Suppose (i) the generation of y in the units {u_1_,…,u_n_} of a population P is described by

$${\text{y(u)}} = \beta {\text{(u)}} \,\text{x}{\text{(u)}} + {\text{w(u)}}
$$

where x(u) is the focal cause—the ‘treatment’ or putative cause, w(u) is a function of all other causes of y in units in P except…, β(u), which may be 0 for some or all u and equals both the difference in y(u) when x = 1 minus when x = 0 (the *individual treatment effect* of x on y for unit u) and the net effect of all interactive/moderator variables.

Note, as just above, that the *individual treatment effect* for unit u is the size of the outcome y(u) when the cause is present minus when it is absent, all other causes held constant. From the proof you see that the *average treatment effect* (*ATE*) in P, which is the expectation of the individual treatment effects, is *Exp(β)*, i.e. the ATE for population P = *Exp(β)*.

Suppose (ii) orthogonality of the treatment with the net effect of the other causal factors, defined thus:

$${\text{Exp}}\left( {{\text{w}}/{\text{x}}} \right) = {\text{Exp}}\left( {\text{w}} \right);\;{\text{Exp}}\left( {\beta /{\text{x}}} \right) = {\text{Exp}}\left( \beta \right).$$

Note that these orthogonality conditions express what is meant by ‘controlling for statistical bias.’ So now we can look to see what follows if statistical bias were to be controlled for in this sense.

Conditioning on x = 1 and x = 0, taking expectations across the equation and subtracting yields

$${\text{Exp}}\left( {{\text{y}}/{\text{x}} = {1}} \right){-}{\text{Exp}}\left( {{\text{y}}/{\text{x}} = 0} \right) = {\text{Exp}}(\beta )$$

Thus we see that *the difference in observed mean outcomes is an unbiased estimate of the study population ATE.* If the average of individual effects of C on E across P is positive then C must have a positive effect on E in at least some individual units in P.

For those readers who are more familiar with the intervention/manipulation or counterfactual approaches to singular causation than with a POE approach I should point out that these all make essentially the same basic assumptions. All three suppose that in most individual cases there will be more than one factor that can causally influence an effect y. All count x as a cause of y for a given individual u if were x to take a different value for u, y would too, supposing all other factors causally relevant to the production of y for u stay fixed except the downstream effects of the change in the value of x. This is the question of whether x has an *individual treatment effect* for u on y. A POE goes beyond this in distinguishing among the other causal factors for y those that are *interactive* with x in the production of y—i.e., factors that affect whether or how much x contributes to y for u—represented by β, and those that are *linear*, i.e. x contributes the same to y for u independent of the values they take for u, represented all told as w.

POEs also suppose that for each case there is a given magnitude for how much x contributes to y, which is a function in part of the magnitude of its interactive variables:

$$[{\text{y(u)}}/{\text{x(u)}} = {1},\beta {\text{(u)}},{\text{w}}\left( {\text{u}} \right){]} - {\text{[y(u)}}/{\text{x(u)}} = 0,\beta {\text{(u)}},{\text{w(u)}}].$$

So all three approaches are in essential agreement in identifying a positive individual treatment effect of x on y for u as the arbiter of whether ‘x causes y in u’ holds.^6^ But, as is commonly noted, we can never directly observe the individual treatment effect, neither with an RCT nor with any other method. What the POE formulation does, via the proof above, is to show exactly what we can learn via an RCT from things we can observe and what exactly must hold for us to be able to do that.^7^

So now, what is so special about RCTs that would make the laudatory but undefined label ‘most rigorous’ appropriate? Three things stand out. First, RCTs estimate an average across quantities that we cannot individually measure, which to me seems astounding, though it may not have all that much to do with rigour. The next do seem the kinds of thing we can expect of a process labelled ‘rigorous’. First is that there is a valid proof that shows why the observed results support a causal conclusion and just what form that causal conclusion takes. The immediate causal conclusion is of the form ‘The ATE for the study population is φ’, and the observed results (the difference between the observed outcome mean in the treatment wing minus that in the control wing) are an unbiased estimate of that. The other is this matter of RCTs being claimed to be the only thing that controls for statistical bias. That has to do with the *premises* of the proof. Any method can be backed by a valid argument if we are allowed to help ourselves to the right premises for it. But not any method is deemed rigorous. So there must be some requirement on premises.

The concern about premises has played a big role in economics in the last two decades, in the ‘credibility revolution’, which ushered in the rise of RCTs, especially in development studies.^8^ Economics has long been dominated by mathematics, both at the theoretical level—e.g. game theory models—and at the empirical—econometric modelling. On both sides the work is sophisticated and intricate, theorems abound. There is no want of formal rigour. But deduction is not enough. The assumptions from which the deductions are made must be *credible*. Consider comments from Joshua Angrist and Jörn-Steffen Pishke (2010) in *The Credibility Revolution in Empirical Economics: How Better Research Design is Taking the Con out of Econometrics*:

With the growing focus on research design, it’s no longer enough to … [label] some variables endogenous [roughly, caused in the system] and others exogenous [caused outside the system], without offering strong institutional or empirical support for these identifying assumptions. (p. 16)

In this they are following on from the work of Edward Leamer (1983), of Christopher Sims (1980), and of David Hendry (1980), especially citing Leamer’s *Let’s Take the Con Out of Econometrics,* where he urges the use of sensitivity analysis to combat ‘the whimsical nature’ of key assumptions. This shows that, as philosopher of economics Julian Reiss (2013) reports, ‘Econometricians often find one another's… assumptions incredible.’ (p. 201).

These economists talk about *credible* premises—ones with strong warrant to back them up. I take that to be the second condition that is required for rigour.^9^ But there’s a further agenda here in the advocacy of RCTs. There is a general distrust of taking almost any of the required substantive assumptions as well warranted. So ‘credible’ here comes to mean ‘either certifiable on the basis of study design alone or established beyond any reasonable doubt’.

With that in hand turn back to the RCT and how it earns the right to be called rigorous. The elimination of statistical bias is represented in premise (ii) in the deductive argument. RCTs are supposed to achieve the required orthogonality in the population P by random assignment of C versus -C to members of P. Great! Except that that only secures orthogonality at the time of assignment. Much can happen after that to confound results. RCTs do have devices to ease that problem—‘blinding’ wherever possible, so that the units in the population, those administering the randomization process, those administering C, those measuring whether E occurs, those doing the statistical analysis, none of these know which units are Cs and which are not.

Great again. But that only eliminates statistical bias due to bias in the sense of prejudice, implicitly or explicitly behaving in ways that affect outcomes by virtue of knowledge of assignment. A lot of time passes between C’s occurrence and E’s, and much can happen in the meantime that affects those units who receive C differently from those who do not that has nothing to do with bias of that kind. Just what these factors might be depends on the specific nature of C and E and of the population P and its setting during the period before and from C to E. What makes it credible in the sense demanded that all these have been guarded against? Further information is needed and what methods can secure that will depend on just what we need to know and again on the nature of C, E, P and the setting. There’s no way to avoid diversity of methods if the premises required for the RCT are to be credible, and the methods are seldom themselves up to securing credibility in the required sense.

That’s all about premise (ii). Now look at premise (i). There’s a lot of metaphysics piled into that. There is first an assumption like that of Donald Davidson (1992) that every singular causal fact falls under some principle or other. The potential outcomes equation (i) represents the overarching causal principle that covers the single units in P.^10^ From its form we see that it assumes something like JL Mackie’s (1974) claim that causes are INUS conditions (insufficient but necessary parts of unnecessary but sufficient conditions) for their effect.^11^ Each term represents a factor sufficient but not necessary for getting a contribution to y^12^ and the salient cause (in this case x) is a necessary but insufficient part of a conglomerate that is sufficient but unnecessary for a contribution. Finally, in (ii) is buried the assumption that there’s a probability distribution over y and its causes applicable to population P.

Now, as to rigour, I don’t see how any of these assumptions can be made credible by methods meeting the requirements identified for rigour. I do not mean to imply by this that we seldom/never have good enough warrant for them. There are many specific cases where we can mount very good arguments warranting the assumptions for that case. But there are no such good cases that I have seen constructed from results or collections of results supplied by rigorous methods alone. Rather they all build on a great diversity of methods, both to establish different facets of the same assumption (as in the pToC discussion below) and to approach the same assumption in a variety of ways relying on a variety of different assumptions themselves.

The lesson that I take to follow from these considerations about premises (i) and (ii) is that RCTs—supposed to be a paragon of rigour—do not after all very often establish the very results they are best at with rigour.

So far what I have been discussing is about what is called ‘internal validity’—about how well the method properly applied secures the targeted conclusion. The notion of rigour I’ve adumbrated implies that rigorous methods will be high in internal validity. In the case of RCTs, granting all the metaphysics in premise i), the lynchpin with respect to rigour and internal validity is orthogonality, which cannot always be banked on even with sound randomisation procedures and successful blinding at all relevant stages. That is half my worry about them. The other half is the familiar worry that goes under the misleading title ‘external validity’.^13^ RCTs at best rigorously secure conclusions of the form ‘The experimental outcome ϕ is an unbiased estimate of the ATE of x on y in population P’. Unbiased means that if you did the experiment endlessly over and over on exactly the same population in exactly the same circumstances the expectation over all those outcomes would be the true ATE. I am not sure how badly you wanted just that result about P in the first place. And often you don’t really want a result about averages in P at all. You want to use evidence from P to draw conclusions about other populations or about individual units in P.

Suppose you want these conclusions about other population or about my focus—what happens in a single case—to be drawn rigorously, in a sense similar to that in which RCTs are rigorous. For that you would need a good argument showing why (granting certain additional premises) the observable results from the RCT coupled with other presumably well-established information you use are evidence of the conclusion drawn. That, as always, is easy if there’s no constraints on the premises. If we are willing to assume that a target population in Birmingham England is sufficiently like a study population in Birmingham Alabama in just the right ways,^14^ then there’s a valid argument that an RCT estimate of the study population ATE in Birmingham Alabama is an unbiased estimate of the ATE in Birmingham England. But we also want the premises to be credible, to be established beyond reasonable doubt or by design alone. Again it is not hard to find cases where we have been willing to take it that this is available. But again, seldom will the requisite premises be secured by rigorous methods but rather by a great diversity of methods that together do the job well enough but cannot lay claim to rigour.

*Lessons.* I propose that what is meant by a rigorous method^15^ in discussions of methods for drawing causal conclusions is one for which a) there is, if not a deductive, at least an argument otherwise warranting the label ‘valid’, why results produced following the method count as evidence for the type of causal conclusion drawn and b) where the premises for this argument, including the relevant results of applying the method, are credible in the sense that they are, or follow from, well-established knowledge or are secured by the design represented in the method protocol.

I have looked at one method highly touted for its rigour.^16^ I have argued that its usefulness is exaggerated. Even with proper randomising procedures and good blinding, it can fail to produce results rigorously because requisite premises can fail to be credible. And it frequently does not go far in telling us what we want to know since that is so often about something other than averages in the population enrolled in the experiment.

There is no reason to think that the RCT is especially open to problems in these regards. It is after all supposed to be the gold standard for one important type of conclusion, and despite all the explicit acknowledgement of the ‘problem of external validity’ it is widely held in development economics and throughout the evidence-based policy movement to be THE method to use to warrant causal claims even if they are about populations other than that enrolled in the study or about individual units. I suggest that what is true of the RCT is true of any single method once you unpack the details of the defence of it: few applications of any one method will meet the standards assumed for rigour and those that do often don’t establish what we want to know.

How does this support diversity of methods? If as RCT advocates suggest all the other methods are even less good at securing results (in this case causal conclusions) why turn to them?

Part of the answer is that this supposition is not entirely right. Many of these other methods can deliver results as rigorously as the RCT in the right epistemic circumstances. That’s because the trick is not in getting a valid argument to back the method but rather in finding premises that are credible in the case at hand. Consider a well-rehearsed example. We may stratify in an observational study to control for confounders. That could provide orthogonality, in which case the same proof as the one I cited shows that the observational study can estimate the ATE for the population in the study. But how secure is the orthogonality assumption? What about those unknown unknowns? In some cases we may have very good reasons arguing that unknown confounders are not responsible for the observed correlations. In others, somewhat good reason. In others, little at all. Just as with an RCT we may often have very good reasons arguing that assignment was genuinely by a random procedure that was not undermined and there was no serious confounding post random assignment. In others, somewhat good reason. In others, little at all. It all depends on what can be called on as already well-established (or that we are in a position to get established in time) that can play the role as premise for this or that method. And that is a highly local matter. Case by case we will have different bits of deemed-secure knowledge that we can press into service as premises for different methods. So better to choose our methods to fit our local knowledge than to despair because we do not have the right knowledge for our most beloved method.

The second part of the answer is that, as I stress in the next section, when we bet on a singular causal claim being true, we are betting on a lot of facts happening. That is generally the case for whatever kind of empirical conclusion we want to draw. Surely it is better to have some evidence about whether these facts will occur or not rather than none at all, even if that evidence cannot be rigorously obtained and all told does not rigorously establish the targeted conclusion.

The third part is that we often must make decisions about what to do for individual units—should we introduce mobile phone nutrition technology in this local Indonesian village clinic, shall we install alley gates to reduce crime in Headington Quarry, shall we prescribe timolol drops for this glaucoma patient? These are both singular and predictive. So we cannot have RCT evidence directly about them, and what it takes to show the evidential relevance of any RCT results we have from elsewhere will often be substantially lacking in credibility. I discuss this a little more fully just below.

Together these three reasons argue that we should have a panoply of methods in our tool bag, not just one golden hammer.

*Moving on.* There is more to be said in favour of diverse methods in the case of causal claims than just the observation that generally it’s better to have a good choice of tools for problem solving rather than trying to adjust your needs to fit the one tool you prefer, which anyway seems obvious on the face of it. One of the reasons I first got so exercised about RCTs and their endorsement for evidence-based policy is that the choice of an RCT to learn about averages in a study population as the instrument to use to make predictions about a different population or about an effect on an individual unit seems perverse. Really, the best way to warrant causal claims in one population is to establish them in another?

Doing that would make sense if the kinds of claims at stake could plausibly be taken to be universal. We can measure the charge on a handful of electrons and to the extent we can take our results on those to be accurate, we’ve learned about them all. But that hardly seems true for the effects of installing alley gates on crime, especially burglaries, as, for instance the UK’s What Works Centre on Crime (The College of Policing) makes clear in its *Crime Reduction Toolkit* since it devotes a section to ‘In which contexts does it work best?’^17^ This is the bulk of what you need to make predictions about the singular claim ‘Alley gates will reduce burglaries in Headington Quarry if implemented in just this (or that) way.’ But notice that what is said in answer has little ‘rigorously established evidence’ as the *Toolkit* itself remarks: ‘Within the review, a variety of contextual factors, which could impact upon the effectiveness of alley gates were discussed… The absence of data in the primary studies meant that these could not be tested.’

This is despite the fact that stress is put on rigour for the *Toolkit*’s overall evaluation that the evidence for positive impact is reasonably strong. They tell us this for instance: ‘**How strong is the evidence?** The review was sufficiently systematic that most forms of bias that could influence the study conclusions can be ruled out.’^18^ (bold orig.) But note the tense of the verb in the conclusion: ‘Overall, the evidence suggests that alley gating has reduced crime.’ This is just what I’ve worried about for a good while. They are studying *there then* to find out about *here now or in the future*. In the next section I sketch a more direct way to tackle this problem that I’ve recently (with co-authors) worked out in detail (Cartwright et al., 2020).

## Causal-process-tracing theories of change: you shouldn’t try to do without them

I think one of the reasons that evidence-based policy advocates despair of learning more directly about the effects of C on E in an individual unit or in the actual population of interest is that they focus too much on C and E and not, as Wesley Salmon (1984) and other advocates of process theories of causality would have it, on the process in between. This provides a very rich source of ways to assess whether C is likely to produce E in that population. Nor does mining this source require adopting a process theory of what causality actually consists in. All it takes is the recognition that, whatever we take causing to consist in, there is almost always a process with several significant steps connecting C and E where each step must happen in turn if C is to produce E.^19^

I recommend then that for evidencing the claim that C will produce E in setting S^20^ we should begin with what we call a ‘causal-process-tracing Theory of Change’ for C and E in that setting (‘*pToC*’ for short) (Cartwright et al., 2020). That shows what kinds of facts must be in place, which in turn allows us to consider what kinds of methods allow us to infer that they will be. This is in the end is going to require a great mix of very different kinds of methods since the kind of facts involved is so diverse. All of which is complicated and hard work. But there’s no getting around the fact that such a process will actually have to occur if C is to cause E in S. You may not choose to model this process and investigate the chances that it can carry through in S, finding it too difficult or too costly or whatever. Nevertheless these are the very matters that will determine whether C succeeds in leading to E, which recommends modelling it and evidencing all the necessary factors possible if you want to get your predictions right.

My focus on the process in between is in no way new. Many of us—and especially the EBM + (Evidence-Based Medicine Plus) movement^21^—have been urging the need for ‘mechanistic’ evidence for a while. What I add here is a model that lays out all the various points where evidence is needed in aid of my general point that good singular causal prediction almost always requires a great mix of highly diverse evidence types and highly diverse methods to secure it. The philosophical foundations of the model will be apparent to readers of *Synthese:* Wesley Salmon’s process theory of causation (1984) coupled with Mackie’s (1974) INUS account and, with respect to causal principles, Donald Davidson’s (1992) arguments for the need for them, Jan Elster’s (2007) account of mechanisms, JS Mill (1848) on tendency laws and Nancy Cartwright’s (2020) discussion of middle-level principles. Methodologically it cobbles together insights from process-tracing and case-study methodology along with realist evaluation.^22^

Perhaps it is worth clarifying before we start that ‘singular’ is opposed here to ‘universal’, ‘fairly general’, ‘widespread under X conditions’, etc. The claims I have in view are ex ante predictions or post hoc evaluations about a given population or a given individual (a specific person or a specific school, state, region, collection of hospitals, etc.) at a given time and place. This does not imply that the effect in question need be what we might think of as individual. It may for instance be about the average improvement in attainment in 12- year-olds in a school district, which will of course involve a number of pupils.

It will be apparent that the conglomerate of methods that will need to be employed to vouchsafe the diverse kinds of evidence represented in a pToC cannot sensibly be argued to rigorously establish an overall causal claim with anything like the degree of rigour that a good RCT can provide for estimating a study population ATE (in the sense of ‘rigour’ adopted here). But there are three special things they can do:

1. They can provide good evidence about effects on an *individual unit*, both ex ante and post hoc.
2. They can provide good evidence for *causal predictions* about a specific population in a specific setting not yet studied without having to rely on claims about how this population in this setting and its causal structure compare with already-studied populations elsewhere.
3. Frederica Russo and Jon Williamson have famously argued that group-comparison statistical ‘difference-making’ evidence of the kind RCTs provide is seldom enough alone to secure causal conclusions. It should generally be combined with mechanistic evidence, where ‘mechanism’ is usually taken to refer to the causal process connecting cause and effect. To the extent that their arguments apply, then pToCs have much to offer even for post hoc evaluation where RCTs are available since they provide a catalogue of evidence types for warranting causal-process/ mechanistic claims.^23^

**Fig. 1:**
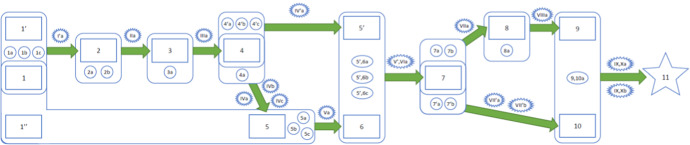
A pToC (from Cartwright et al., 2020)

Figure 1 provides an example constructed by me and co-authors (with Tamlyn Munslow in the lead) of a pToC for a real-life casual process set in motion in Indonesia in North and East Jakarta, Surabaya, Pontianak and Sikka in 2013: mHealth, a programme that introduced innovative mobile phone applications to support nutrition outcomes where there were issues with manual growth monitoring. The one I’ll focus on is that incorrect categorisation of children’s weight as normal was leading to children being missed from being offered nutrition support services. In 2013, World Vision Indonesia, working with MOTECH Suite, designed a mobile phone-based application to address this among other challenges hindering nutrition service delivery in Indonesia. (The key for Fig. 1 is too long to reproduce here in its entirety. I include the key for the first couple steps below. For the entire thing, see the original paper.)

Like the defence of the RCT, the structure suggested for a pToC makes both the Davidson-like assumption that, at least where prediction and warranted retrodiction are possible, causes do not produce their effects willy-nilly but do so in accord with principles that hold more widely than the single case (though those principles may not describe the factors in the same way that we do) and the Mackie-like assumption that causes are INUS conditions.^24^ Here briefly are the kinds of features that appear in the pToC:

1. The significant steps by which the programme is to produce the outcome.
2. The casual principles by which each step is to lead to the next. The principles are not actually pictured in the figure but are meant to be listed alongside. They should be a big help in figuring out the next three ingredients.
3. Support (interactive) factors. This supposes, following Mackie, that the causes that are highlighted in typical causal principles are seldom if ever enough on their own to produce the indicated effect. Rather to do so, they need to operate in tandem with other factors—support factors. In Fig. 1 all the factors that together are taken to be enough to produce the effect are enclosed in a circle. When necessary support factors are missing and cannot be substituted for, the causal process cannot carry through as expected.
4. Derailers. These are anything that can intervene and stop or diminish a process once it is in train. These are represented by jaggedy ovals.
5. Safeguards. Safeguards can be thought of as ‘walls’ that prevent derailers from intruding. When safeguards cannot be implemented against every likely derailer, we should be wary of predicting programme success.

The pToC for how the programme evolves provides a framework for categorizing the kinds of evidence that, all told, can make effectiveness predictions reliable. This includes evidence for

- The causal principles that are supposed to work at each step and that they can be called into play and at the right time
- At each step the support factors will take appropriate values to allow that step to produce the next
- At each step derailers will be absent or guarded against.

There is no rigorous way to establish these separate kinds of evidence nor to assess what they amount to all told. The kinds of information that are helpful are varied, so too are the methods that can provide evidence for them. What is clear is that, if you want to avail yourself of this additional evidence—as you should—you will have to employ a great variety of different methods and a good deal of judgment. This kind of pToC, well done and well-supported, can yield reliable predictions about programme success. It can do so even though none of the pieces, nor their overall impact, are rigorously supported. Conversely, I have never seen any way to do the same job rigorously.

Let’s look at the first couple steps in this pToC to illustrate. Here is the key for them:
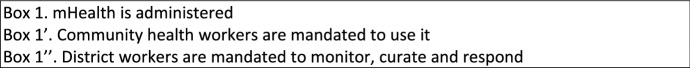


**Principle 1,1’-2**: **Health workers tend do what they can in their clients’ best interest.**

Support factors:

- Community health workers have the capacity to use mHealth.
- Community health workers agree that using mHealth is good for their clients
- Mothers and children attend the community health clinic on a regular basis

Derailers:

- I’a. External pressure to not perform the task or other priorities prevail

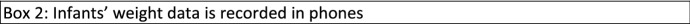


Principle 2–3: mHealth technology does accurate calculations of growth status

Support factors:

- 2a. mHealth technology is well designed for the job
- 2b. Community health workers input the correct data in the correct format

Derailers:

- IIa. Technology fails to operate

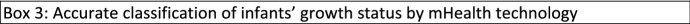


The two causal principles invoked here are typical for this kind of enterprise. The causal principles that programme design and prediction rely on are generally ‘middle or low level’—neither high theory meant to obtain everywhere nor so narrow as to hold almost only for the case at hand, and they often come in the form of generics, without quantifiers or explicit range of application. They are what JS Mill (1848) labels ‘tendency principles’. They frequently describe dispositions of individuals or institutions that are widespread but don’t appear everywhere, and often where they do obtain they need the right setting or stimuli to be called into play.^25^

The two are quite different with respect to the kinds of methods needed for warranting them. The first, that perceiving an action to be in their client’s best interest disposes health workers to do that action, is common knowledge. The issue is not establishing it but rather establishing that it will be called into play in this case and then not be overwhelmed by competing dispositions. This places the bulk of work on warranting the support factors and derailers that are identified and evidencing their presence or absence. This is a good reminder that for making and warranting singular causal claims like these effectiveness predictions not all the methods to be used are taught in science classrooms or methodology courses. ‘Call on common knowledge’ is an important tool in our methodology tool bag.

The second principle employed does take warranting. It says that inputting a child’s weight and other information into the phone app will produce an accurate judgment about the child’s growth status. The methods to warrant this include a great deal of theorical derivation and use of local knowledge about what the right answer is and about the mechanism by which the app does the calculation as well as trials under various conditions that it works properly, and more. Not all these methods need be employed in the immediate defense of the expectation that the causal process will carry through start-to-finish. Sometimes it can reasonably be taken on faith that they have been employed, and employed properly, by someone else somewhere else. Still that should not blind us to the recognition that they (or a good substitute for them) are all necessary if the prediction is to be genuinely warranted. Some are of immediate importance though. It would generally be foolish to send phones that have been shipped from abroad out to community health centres without some method that checks that they work properly.

Beyond the principles, a host of other things pictured in the pToC need to be considered—all the support factors, derailers and safeguards. Why can you assume that the community health workers are able to use the apps? Perhaps because of background knowledge that they are accredited nurses and also use equipment like this all the time and that they have the time to use the phones properly. But in Indonesia that was not the case. The community health workers were for the most part untrained volunteers. Perhaps then because there are records that they have participated in a training course or from questionnaires or from test results. And so forth.

So, to evidence a singular causal claim I propose that you first construct a plausible pToc for the targeted cause/effect pair in the setting under consideration. The pToc should contain the 5 types of information I listed above. It is plausible when (a) each step is connected to the previous by a casual principle for which there are reasons in support of the assumption that that principle *could* operate in that setting, and (b) the support factors and derailers at each step are appropriate to that causal principle. The pToC then shows you what facts should be evidenced as part of a large body of evidence about the overall cause/effect claim. You then want whatever evidence is possible, gathered by whatever methods are appropriate to it, that each and every support factor and requisite safeguard is (or was) likely to be in place and each and every derailer that is not safeguarded against is (or was) likely to be absent. That’s a lot of evidence to gather. But these are after all the facts that have to obtain if the cause is to lead to the effect in the way pictured, and to the extent that any have no evidence in their favor, to that extent our case in favour of the cause/effect claim is weakened. Would that it were easier. But there are few singular causal claims that can be supported in any less demanding way.^26^

I advocate construction of the best pToC possible to use as a guide for figuring what facts count as evidence for or against the overall prediction that C causes/will cause E in S. But there are several cautions to note about its use.

a. How do we come up with support factors and derailers for the pToC? The causal principles suggest what’s needed. That’s why it is important to have principles that we have good reason to suppose apply and can be triggered in the setting and to express these as concretely (‘low level’) as possible. In the Indonesian setting there was good reason to suppose that community health workers were positively disposed to act in the children's interests and also that World Vision Indonesia and MOTECH had done a proper job in vetting the phone app technology. Supposing we are relatively confident that the causal principles can operate in the setting, it then takes a great deal of local knowledge to figure out what supports and derailers would be there. 1b.—that the health workers believe that using the technology is in the children’s best interests—seems an obvious necessity. But 1.a. and 1.c., which are in a sense equally obvious, might not have been thought of by programme designers accustomed to trained staff and parents who don’t face severe difficulties (including cultural views about health provision) in getting children to clinics.

The presence of the intermediate steps and support factors and the absence of the derailers represented in the pToC counts as evidence only if these genuinely are necessary intermediates, supports and derailers. That is, these are evidence only supposing the pToC is right. It is not surprising that we need to make some suppositions. The reason we need arguments that *show* why a given fact is evidence for a given hypothesis is that facts don’t come labelled as evidence. Assumptions are always needed to turn them into evidence—like the assumptions in premises (i) and (ii) in the RCT argument that turn facts about observed mean outcomes in the treatment and control groups into evidence for the size of the study population ATE. But supposing that a given pToC is correct can seem a very big assumption.

This need not be the case though. Part of the point of breaking the envisaged process into small steps and trying to see what principles would need to operate at each step is to allow us to invoke not just general but local knowledge of what those principle might amount to in the setting and what might be needed—e.g., knowing that the health clinic workers are untrained volunteers from rural villages, helps in recognising that it is important to explicitly note that it is necessary that they be able to use the app technology properly.

The starting point for a setting-specific pToC of the kind I urge will often in cases of policy interventions be a more general ToC constructed by the programme developers who have thought through why they expect the programme to work and how. A general ToC for a programme that is hoped to work in more than one setting will usually assume causal principles expressed at a more middle, abstract level—e.g. ‘People respond to incentives’— with concomitantly abstractly expressed supports and derailers^27^ —e.g. ‘X is perceived as an incentive’. To be most useful the pToC for the setting should use far lower-level principles and use far more concrete concepts. In general we would expect the local pToC to be constructed and refined recursively as more local information is available or sought out.

b. It is important to keep in mind that one may have excellent evidence for the singular causal claim that C will cause E in S and indeed the process actually carries through as envisaged, but yet E may not obtain. An anonymous referee has raised worries about a problem that Gerhardt Hesslow (1976) made apparent with his famous birth-control-pills example and that Cartwright (1989) called ‘causes with dual capacities’ and others since^28^ have labelled ‘masking’, where the same cause triggers different processes whose outcomes cancel each other. But matters are in a sense worse. A pToc like this only traces out one causal pathway from one cause, and many other causes can influence the same outcome—all those factors represented by ‘w’ in the POE of Sect. 3—even to the point of producing an opposite effect to the one hoped for. To address that problem one needs a far fuller local causal model. (For a discussion of what form such models might take and how these can be used to show the relevance of standard types of evidence for warranting singular causal claims see Cartwright, 2017.)

Equally important, as another referee points out, one may very well establish, properly with a great body of diverse evidence, that C caused an unwanted E in S, but that does not mean that removing C-type events is a good strategy for preventing future E’s. Often what’s needed is a structural change to make it difficult for C-to-E type processes to occur. This is what Eileen Munro, author of the UK (2011) *Munro Review of Child Protection* argues about child welfare. When a child welfare tragedy occurs, she explains,

[t]he standard response is to hold an inquiry, looking in detail at the case and trying to get a picture of the causal sequence of events that ended in the child’s death…We are tracing a chain of events back in time to understand how it happened.… (Munro, 2004, 381)

Instead, she urges, ‘Child protection is a systems problem.’ And she quotes the US National Academy of Science’s *To Err Is Human: Building a Safer Health System* (Kohn et al., 2000):

The focus must shift from blaming individuals for past errors to a focus on preventing future errors by designing safety into the system. (21)

c. I have offered a catalogue of diverse evidence types. I do not here hazard suggestions about how then to assess the sum total of evidence gathered along with estimates of the accuracy of the pToC that justifies these as evidence and estimates of the accuracy of the evidence claims themselves in order to arrive at an overall verdict on whether C will in fact cause E in S. Since these pToCs involve a kind of process tracing, standard advice from there can be of help, e.g. thinking in terms of van Evera, 1997’s classic four kinds of evidence: Smoking Gun, Hoop test, Doubly Decisive, and Straw-in-the-Wind. For instance, strong warrant that a necessary support factor was/will be missing with nothing to substitute for it counts strongly against the causal claim—it fails the Hoop test: a test that cannot be failed if the claim is to be judged true. Also there has been a surge of recent Bayesian-inspired work, for example that of Befani (2021).

d. The mention of process-tracing evaluation methods brings to the fore an important kind of evidence made much of there as well as generally in theories of warrant, a kind that is not directly represented in a pToC. A pToC lays out facts that are evidentially relevant to directly confirming or disconfirming the hypothesis in view. It does not say anything about evidence that could count for or against alternative hypotheses about what will happen if C occurs, which we would also very much like to have. This kind of evidence does enter to some extent in the consideration of support factors and derailers, as Munslow (2021) argues. We do after all suppose the cause is actually introduced so that alternative hypotheses involve alternative causal processes ensuing. Given plausible causal principles, what prevents the next step following in train in the way hypothesised would be the absence of necessary supports or the presence of unguarded derailers.

e. One might wonder how to use a setting-specific pToC to learn about what might happen elsewhere. I don’t have much to offer here. This is a general question for case-study and single-case process-tracing research so we can look to these two areas for guidance, for example the classic George and Bennett (2005), Guala (2010), Ruddin (2006), Steel (2008) or Yin (2012). What I think not likely to work so well is trying to move cross-wise from one specific setting to another. As mentioned, a really useful setting-specific pToC will generally be couched in very concrete terms in order to make clear just what really is required in the local circumstances. These very specific factors may not be what matters elsewhere. A way to proceed instead would be to ‘abstract’ trying to see in virtue of what more general descriptions the causal principles and factors play the role they do in order to construct a more middle-level pToC, then look to see what would constitute a concretization of that in the new circumstances.

f. An anonymous referee asks about how pToCs can contribute to questions about the size of an effect in a new setting. I can say at least this. As we see from the proof in Sect. 3, effect sizes are a function of the distribution of support factors (interactive variables). For simplicity I’ll suppose we are dealing with yes–no variables only since this is easier to explain. Consider a case where we have good reason to suppose all the requisite support factors for all steps can be present and all derailers absent. If that held for every unit in the setting population, the effect would be achieved in every unit. But generally some of these will be present in some individuals and not in others. The effect will not be achieved for any individual that is missing any necessary support factor^29^ or suffers any unguarded derailers. I haven’t mentioned this before but very often there will be different combinations of support factors that combine to be sufficient for a given step to lead to the next. If only you could put a probability measure over this gigantic event space of supports and derailers, you could sum the probabilities of all the ‘positive’ combinations and thus calculate the portion of the population for which the effect will be achieved.

Before concluding, let me in response to a referee’s query turn to the metaphysics behind a pToC. One of the nice things about a pToC is that it makes only very common assumptions about causality that can hold on almost any metaphysics of causation.

I should note at the start that though there is a close connection between metaphysics and method it is not so close that metaphysics dictates method. For instance, a counterfactual characterisation of causation requires that there be the right kind of truth about what would happen if the cause were absent, other causes held fixed. But this does not imply that the only evidence that such a counterfactual claim is true in a given case is by a direcrt frontal attack: seeing its actually obtaining in the given case, contrary to possibility, or seeing its actually obtaining in a case that is ‘identical’ to the given one. GEM Anscombe (1971) claimed she could *see* the cat lapping up the milk just as she could see the cat and the milk themselves, that is, she could see the cat causing the milk to disappear. If a true singular causal connection requires a counterfactual truth, then seeing the cause produce its effect must count as evidence of the counterfactual truth—it is an indirect way to establish that the requisite counterfactual is true. Or, closer to our pToCs, suppose C occurs and E occurs but none of the support factors necessary for C to produce E are there or there is good evidence that serious derailers operated. Again, if C causes E just in case the requisite counterfactual holds, then we have ample evidence that the requisite counterfactual did not hold since the casual claim can’t be true if required support factors are missing.

This is one of the things I like about pToCs. They make essentially only three fundamental assumptions about causality and each is consistent with all the standard accounts of causality that I know and some or all are actually presupposed by many of these accounts.

First the pToC approach supposes that where C at one place and time contributes to producing E at another place and time, there is a causal process with steps in between connecting the two. We need not even suppose one way or another whether that process is continuous or discrete. This assumption need not hold universally—perhaps it fails in some kinds of case, as is sometimes argued about wave-packet reduction in quantum mechanics. No matter. In these cases it simply turns out that a pToC is not a useful tool for cataloguing evidence.

Second it supposes that the effect cited at step n that was supposed to have been partially caused by the factor cited at n-1 will not usually be sufficient on its own for the effect cited at n + 1 which must figure as a cause of n + 2. It needs the presence of support factors and the absence of derailers (or something that stops the derailers interfering). That is just the familiar ‘insufficient but necessary part of a sufficient condition’ for a contribution that we know from JL Mackie. This assumption too fits with all the metaphysical accounts of causality that I am familiar with.

Third is more metaphysically controversial. The pToc as we have constructed it supposes that the singular cause/effect relation that occurs at each step does so in accord with some general causal principle. It is neutral though about whether this is a governing principle or just descriptive of what does in fact often cause what. It is equally non-committal about what ‘causes’ in the causal principle amounts to, for instance is it a kind of very active pushing the effect into existence or instead just a report of some complex regularity of the kind we see in standard Humean approaches or in some probabilistic theories of causality?

I personally resisted this assumption for a long while. (See for instance my discussion with Donal Davidson on the Philosophy International Donald Davidson Videos.) But that was before I started to think in terms of smaller steps. One of my favourite examples is of the Rube Goldberg machine where flying a kite causes a pencil to be sharpened. That cause/effect pair does not fall under any general causal principle. BUT—the process in between can be broken down into steps each one of which does fall under a familiar causal principle. Indeed that is why we can be so sure without building it that the machine would operate as expected. Nevertheless, we needn’t make the strong assumption that all singular causings occur in accord with a principle. In cases where some one or another of the steps between C and E do not, then that’s a case where we cannot use a principle as a tool for figuring our what support factors and derailers are likely to be and hence for figuring out what kinds of evidence to gather about whether that step will carry through.

## In sum

When you bet on a singular causal claim—C causes E in setting S—like the policy effectiveness predictions used here for illustration, you are betting that the causal process connecting C and E will carry through successfully in S. You are betting on that whether your defence for doing so is that a similar process must have carried through elsewhere because there is good evidence (perhaps from RCTs) that C has caused E elsewhere or because you have evidence that directly supports that prediction about S or you are just betting without much warrant.

The pToC for the process lays out what must be in place for it to carry through. Again, whether you detail the features in the pToC and provide warrant about whether they will be present or not, their presence or absence will determine success or failure. Given that you are betting on success, evidence seems in order. And producing that evidence, whether done by you or someone else somewhere else, requires a panoply of diverse kinds of methods. There’s no way to warrant singular causal claims with only a single method—or a handful of them.

Few of these methods will be able to secure the information aimed for rigorously in the sense of ‘rigour’ I’ve identified for RCTs. And there certainly is no rigorous way to figure what all the information together amounts to. This is not to recommend giving up on rigour when you can have it. Just do not overestimate what it can do for you—and definitely don’t let it dictate what methods can be used.

The logo for this paper^30^ is the South African Jacana bird. Rigorous results are like short, sturdy sticks, good for jobs where a small rigid piece will help. But relying on rigorous support for the products of science is like resting your precious bird’s eggs on a heap of sticks rather than, as the Jacana birds do, collecting a hotchpotch of leaves, sawweed, hornwort pondweed, saliva and feathers and weaving them together, with the sticks, into nests sturdy enough to support their eggs, even though the nests float on water, as Otto Neurath taught that all of our knowledge claims do.

## Footnotes

1 An anonymous referee here points out that it is well-known and thus not worth rehearsing that RCTs cannot provide rigorous evidence for singular causal claims. But that is not my point. I argue that RCTs often cannot rigorously establish even the kind of conclusion they are ideally suited to: an unbiased estimate of an average treatment effect is a study population. So the very paradigm of rigout often fails at its task. This makes it far less damming when we note that other methods for establishing other conclusions may also fail to provide rigorous support. Also, the very kinds of information it takes to secure rigour for the unbiased estimate are just the kinds that I recommend in my catalogue for evidencing singular claims.

2 https://seniorsecondary.tki.org.nz/Science/What-is-science-about. Accessed 10 October 2020. (Ital. added).

3 The Abdul Latif Jameel Poverty Action Lab. https://www.povertyactionlab.org/.

4 https://www.nobelprize.org/prizes/economic-sciences/2019/press-release/. Accessed October 10 2020.

5 A portion of what I say in this section will be familiar. See for instance Deaton and Cartwright (2018). But I want to assemble these points carefully together to show how they argue in favour of diversity of method. Also, I think that the complex strategy I propose in Sect. 4. will be more palatable against a clear background of the failings of even the best of simple one-method strategies.

6 For more on the relations between (formalised) counterfactual approaches and POEs see Galles and Pearl (1998) and Halpern (1998). Judea Pearl (2015) remarks that these ‘have given a complete axiomatization of structural counterfactuals, embracing both recursive and nonrecursive models... They showed that the axioms governing recursive structural counterfactuals are identical to those used in the potential outcomes framework, hence the two systems are logically identical—a theorem in one is a theorem in the other’ (151).

7 No doubt one can begin directly from a manipulation or counterfactual approach to mount an argument showing why the results of RCTs can count as evidence about ATEs. But in the end it will have to adopt something akin to a POE because randomisation is a probability notion so it requires an event space and a measure over it, and what randomisation secures is orthogonality (as opposed, for instance, to balance in the single run), so it will have to end in some claims like ‘unbiasedness’ about what happens ‘in the long run’.

8 And not just in economics. Consider this from the very first line of a very recent Princeton Press book on integrating theory and empirical results by a team of political scientists (Ashworth et al. (2021)): ‘The rise of formal theory and the credibility revolution are two of the great developments in social science over the past half century.’ (p. 1).

9 Obviously not every instance of an RCT can be expected to have credible premises. So the requirement needs to be put more carefully. I think the generous way to render this for those who maintain the rigour of RCTs is to modify the claim: RCTs have a type of premise that can be made credible in the very design of the experiment (i.e. by random assignments and blinding). You see that I dispute even this.

10 Note that some of the terms in (i) can be functions of u that take value zero for some u’s. So this does not assume that all units are vulnerable to the exactly the same causes.

11 This was queried by an anonymous referee. You get from Mackie’s formula to the POE form in the usual way for going from bi-variate to multivariate forms, turning ‘or’s into pluses and ‘and’s into multiplication. The notion of sufficiency than becomes ‘sufficient for a non-zero contribution to the effect’.

12 Recall that W can consist of many terms that themselves have internal structure like the first term.

13 Misleading because it suggests that there is something about the result itself (which recall is of the form ‘ϕ is an unbiased estimate of the ATE of x on y in population P’) or the method (the RCT) by which it is achieved that makes it strong evidence for some fact about some other population Q. (The one exception I can think of is when the standard RCT methodology is combined with random sampling of the study population P from Q, in which case the method does allow inference to ‘ϕ is an unbiased estimate of the ATE of x on y in population Q’.).

14 For example, the two populations can be correctly represented by the same POE and the requisite support factors have the same probability distribution in the two.

15 Or, in response to the concerns in footnote 9, the rigorous application of a method.

16 Or, for readily admitting rigorous applications.

17 https://whatworks.college.police.uk/toolkit/Pages/Intervention.aspx?InterventionID=37&src=%23filter%3D1.

18 Ibid. Bold orig.

19 For Salmon these are points of interaction of the process with other processes.

20 Or *has* produced it. The method is indifferent between prediction and retrodiction (e.g. for post hoc evaluation). I talk of prediction throughout for ease of expression.

21 https://ebmplus.org/.

22 And probably more that isn’t right to the fore right now. For readers not familiar with realist evaluation I recommend Pawson and Tilley’s classic 1997 book.

23 For this purpose the pToc approach provides a nice supplement to Chapter 5 of Parkkinen et al. (2018) (‘Gathering Evidence of Mechanisms’) which outlines steps for gathering evidence but does not detail what diverse kinds of evidence should be gathered.

24 But not all INUS conditions for the effect are diagrammed, only those in the process from the cause under consideration.

25 Jon Elster (2007) calls these ‘mechanisms’. For more about middle-level principles see Cartwright (2020).

26 One alternative is support via deduction from a general causal principle that holds exceptionlessly. Gravity is a good example because it seems it cannot be shielded against. A mass will always cause an attractive force on another mass even if the other mass never moves due to the action of other causes. And being born will always lead to dying. But unlike these, many of our most useful casual generalizations are ceteris paribus, with exact conditions left unspecified.

27 Where these are suggested at all. Programme developers are now widely urged to produce ToCs for their programme but not many are seriously detailed.

28 See inter alia Illari (2011).

29 And no substitutes occur.

30 Like that for the forthcoming book that will take up similar themes, *The Tangle of Science: Reliability Beyond Method, Rigour, and Objectivity*. (Cartwright, Hardie, Montuschi, Soleiman and Thresher).
